# Supplementary material for: Are carbon emissions trading and green financial instruments synergistic? -Comprehensive quantitative research based on content analysis
Source: PLoS One. 2024 Mar 7;19(3):e0298601. doi: 10.1371/journal.pone.0298601 (PMC10919601; doi:10.1371/journal.pone.0298601)
Supplement: S1 Data — (ZIP) [file pone.0298601.s001.zip › Data/地级市Z_F工作报告.zip!/║┌┴·╜¡/║╫╕┌╩╨/└┤╫╘╣1⁄2╓┌║┼í╛┬φ┐╦╩2╛▌═°í┐_2005.docx]

各位代表：

我代表鹤岗市人民政府向大会作政府工作报告，请予审议。

 一、2004年工作回顾

过去的一年，我们在省委、省政府和市委的领导下，紧紧抓住振兴东北等老工业基地战略的历史性机遇，不断深化各项改革，加大抓经济、抓发展的力度，强力突破重点难点问题，整体经济快速健康发展，地区生产总值达到104.9亿元，比上年增长12.2%，提前一年实现了突破百亿元的任务目标，圆满完成了市十三届人大三次会议确定的各项任务。

——全面落实惠农政策，农业发展形势喜人。深入落实农村税费改革各项政策，“一免两补”及时兑现到位，农民生产积极性空前高涨。农作物播种面积增加36.9万亩，粮食总产量达到8.64亿斤，增长38.5%；农业增加值达到8亿元，增长14.3%。畜牧业发展水平迅速提升，规模化、工厂化养殖小区达到20个；奶牛存栏1.03万头，增长5.5%；生猪饲养量42万头，增长13.5%；牧(渔)业增加值达到2.09亿元，增长20.8%，占农业增加值的26.1%。农业基础设施建设取得新成果，投资2.5亿元完成了绥滨松花江堤防加固等防洪治涝工程、黑龙江中兴护岸等国土防护工程、绥滨傲来渠首等灌区改造与节水灌溉工程，抵御自然灾害的能力进一步增强。

——工业发展步伐加快，速度和效益同步增长。工业经济保持快速运行，实现增加值26.5亿元，增长20.9%，是近年来增幅最大的一年；实现利税5.8亿元，增长1倍。地方工业继续快速发展，实现增加值7.2亿元，完成利税1.6亿元，分别增长16.4%、20.9%。骨干企业的拉动作用日益突出。鹤矿集团原煤产量增加186万吨，增长12.5%；鹤电公司发电量增加3亿度，增长12%；宝局工业增加值实现3.89亿元，增长14%；东兴集团、华隆玻璃、工农洗煤、三精千鹤、鹤鸣米业等骨干企业都实现了大幅度增长。技改项目进展顺利，发展后劲进一步增强。实施了20个重点技改项目建设，完成投资4.3亿元，其中鲲鹏30万吨焦炭、万禾元油脂、竣丰洗煤等地方超千万元的项目8个，当年新增销售收入1亿元、利税1200万元，是开工项目最多、投资额度最大、经济效益最好的一年。

——积极实施大项目牵动战略，老工业基地振兴开局良好。按照“建设两大基地、加快六业发展”的总体任务，积极争取上级支持，大力招商引资，重点项目开发建设取得重大进展。投资20亿元的鹤岗电厂二期工程开工建设，投资12.1亿元的采煤沉陷区治理二期工程开始启动。投资7.96亿元的鸟山煤矿建设项目获国家批准，投资1.9亿元的100万吨稻谷精深加工是东北地区唯一获国家批准的粮食加工项目，投资9.3亿元的10万千瓦矸石热电厂项目、投资9800万元的矿井水净化项目获省批复，这些项目今年有望开工建设。年产120万吨燃料甲醇项目获省批准，正在招商引资。固定资产投资达到26.5 亿元，增长15.3%。

 ——招商引资成效显著，对外开放进一步扩大。大力开展“招商引资年”活动，加大项目招商力度，招商引资到位资金9. 8亿元，增长11.7%，引进项目274个。对俄经贸合作快速发展，项目合作向纵深推进。完成了4000公顷土地种植、4200平方米房地产开发和黄金开采等七个合作项目。对外贸易大幅增长，进出口总额完成2939万美元，增长139%；口岸运行态势良好，出入境货物17.9万吨，增长25.8%，过客1.9万人次，增长59.1%。

 ——城乡基础设施建设加快，承载功能进一步增强。住宅建设开工55万平方米，竣工6 0万平方米；康居工程完成10.9万平方米。建设通乡公路63.7公里，在全省率先争取到通村公路建设试点，投资200 0余万元，建设通村公路192.7公里。新建、扩建黄金路等10条城市主次干道；辟建了1.7万平方米的振兴广场，完成了“十小工程”建设和3个公园改造任务。投资320万元建设了城区防洪工程，部分地区防洪标准达到20年一遇。城市亮化、绿化、硬化又有新进展，专业植树3.62万棵，义务植树30.4 万棵，大树进城2095棵，超额完成计划；亮化工程继续推进，在24条道路安装路灯581基、6 80盏，在3条道路安装景观灯138基、9835盏；完成了1.9万平方米的硬化任务，市区主次干道基本消除了裸露路面；城市净化力度加大，实现了垃圾晨清扫、夜清运、街道全天候保洁。

 ——非公有制经济加快发展，第三产业稳定增长。以培育“三型”企业大户为载体，提高了非公有制经济的发展水平。民营经济实现增加值21.8亿元，增长16%；实现税收1.27亿元，增长13%；新上100万元以上项目49个，投资5.35亿元。第三产业发展加快，增加值达到32.5亿元，增长12.4%。旅游业又有新发展，接待市外游客20多万人次，旅游收入达到7000多万元，增长14%。

 ——财政收支增长较快，金融运行平稳。财政收入完成8.4亿元，增长26.4%(扣除免收农业税因素)，总财力创历史最好水平。财政支出完成12.1亿元，增长15.4%，保证了农业、教育、科技等重点支出，增加了对产业结构调整、基础设施建设和社会保障的支出。加强了财政预算资金的预算执行审计，提高了资金使用效益。金融机构各项存款稳步增加，信贷资产质量提高。存款余额134亿元，比年初增加13亿元；贷款余额115 亿元。

 ——各项改革深入推进，发展活力日益增强。免除农业税改革试点成效显著，“一免两补”落实到位，免除农业税8000余万元，发放粮食直补资金8200万元、良种补贴522万元，乡镇机构、乡村财政财务管理、农村义务教育体制等配套改革基本结束。完善城镇社会保障体系试点工作实施顺利，实现“并轨”29923人，发放经济补偿金24975万元。企业改革不断深入，鹤矿集团加入省龙煤集团；境内佳铁分局所属教育、医疗机构的接收工作顺利完成，鹤矿集团所属学校移交地方的工作正在进行。中小学人事制度改革全面启动，实施了中小学教职员工聘用制。粮食流通体制改革取得新进展，完成了企业清产核资和用工制度改革。财政改革实行了国库集中支付制度，推进了政府采购，强化了财政监督。农村信用社改革完成阶段性目标，实现增资扩股2.18亿元。

 ——城乡居民收入提高，人民生活显著改善。城镇居民人均可支配收入达到6532元，增长14.3%，农民人均纯收入达到3177元，增长9.7%。城市人均居住建筑面积达到17.55平方米，农村人均居住面积达到21.42平方米，高于全省平均水平。城乡居民储蓄存款余额达到110 亿元，比年初增加5.8亿元。城乡消费市场活跃，实现消费品零售总额25.9亿元，增长10% 。弱势群体利益得到保障，发放养老保险金1.2亿元、失业保险金405万元，10万名低保对象得到基本生活保障，实现了应保尽保。城镇登记失业率控制在4.3%以内，有25155人实现就业和再就业。高校毕业生、复员转业军人的就业、安置工作也取得了较好成绩。农民工工资拖欠问题得到较好解决。

 ——社会事业全面进步，精神文明建设不断加强。科技项目立项61 项，其中列国家和省计划项目23项，争取专项投入468万元。“两基”教育成果进一步巩固提高，教育管理体制不断完善。办学条件进一步改善，新建校舍3.38万平方米。学校布局进一步调整，提高了教育资源的优化配置。普通高考取得了全省文科平均成绩第一名、理科第二名的佳绩，连续九年高考升学突破千人。职业教育进一步发展，开展职工岗位培训和再就业培训7.5万人次，农村转移劳动力培训1.7万人次。师专基础设施建设进一步加强，教育教学水平不断提升。疾病防控体系和医疗救治体系不断完善，初步建立了突发性公共卫生事件应急机制，重点加强了疾病防控、农村卫生和卫生环境建设，投资2710万元建设了疾病预防控制机构、传染病专业防治机构和医疗急救机构，疾病疫病预防控制体系粗具规模。积极推行计划生育综合改革，稳定低生育水平，人口出生率5.32‰，自然增长率0.51‰，符合政策生育率达到99.1%。文艺创作繁荣活跃，参加省级以上文艺赛事8次，获77项奖励，群众文化活动丰富多彩。广播电视大力改革节目形式和内容，人民群众的业余文化生活更加丰富。全民健身运动日益普及，竞技体育有新提高，参加全国比赛获金牌7枚，参加全省比赛获金牌5 4枚。深入开展严打整治斗争，强化综合治理，社会治安形势进一步好转，刑事案件发案率比上年下降6.7%，人民群众对社会治安满意率达到91.2%，居全省第一位。安全工作得到加强，全面落实安全生产责任制，深入开展安全专项整治，煤矿安全生产体系进一步完善，交通、消防等各项安全生产形势明显好转，我市获省政府安全生产责任制考核优秀单位，地方煤矿安全生产获省政府长效机制优胜奖，消防安全连续九年无重特大事故。信访工作合力攻坚，形势稳定，进省进京越级访同比下降。民族、宗教、外事、侨务、国防、人防、双拥、民政、人事、档案、老龄工作等事业也取得了新成绩。

 各位代表，2004年是不平凡的一年，是我们克服困难，振奋精神，抢抓机遇，奋力拼搏，经济社会快速健康发展的一年。成绩来之不易，是省委、省政府和市委正确领导的结果，是全市人民、各经济单元共同努力的结果，是中省直部门和社会各界大力支持的结果。在此，我代表市政府，向全市广大工人、农民、知识分子和各级干部，向驻鹤部队、武警官兵和公安司法干警，向关心和支持鹤岗市建设和发展的社会各界朋友，表示崇高的敬意!向人大、政协、各民主党派、人民团体对政府工作的信任和支持，表示衷心的感谢!

 回顾过去一年的工作，还存在一些困难、问题和矛盾。一是重点项目建设没有大的突破。市县区属地方项目开发建设数量少，能够控制资源、占领市场、具有较强竞争力和较高效益的大项目少。二是积淀的问题多、困难大。长期的计划经济体制积淀了很多历史遗留问题，企业转制后大多数拖欠职工工资、养老保险金、失业保险金和经济补偿金，政府及其机关企事业单位拖欠工程款数额巨大、失业人员多、就业岗位少、社会贫困面大等问题还没有得到很好解决。三是体制性、结构性矛盾突出，经济实力不强，经济结构单一；经济总量小，地方经济总量更小，解决困难、抵御风险、支持发展的能力弱。四是思想观念滞后。同省内外发达地区相比，我们在思想观念上还有很大差距，一些先进发达地区早已解决的问题，我们还依然存在，严重制约重点难点的突破，严重影响经济社会发展。这些问题需要在今后的工作中认真加以解决。

二、2005年经济社会发展的主要任务

 2005年是全面实现“十五”计划目标，谋划“十一五”计划的重要一年，是深入实施老工业基地振兴战略，加快经济社会发展的关键一年。按照市委工作会议确定的工作思路，今年政府工作的总体要求是：认真贯彻落实中央和省委、省政府的战略部署，牢固树立和全面落实科学发展观，以加快发展为主题，以推进老工业基地振兴和资源型城市转型为主线，围绕增加财政收入、富裕人民群众、构建和谐社会，抓经济，搞建设，谋发展。提高原煤产量，加强煤矿安全，加快煤炭转化，重点抓好煤转电和煤化工。提高粮食总产，增加水稻面积，扩大畜牧规模，加强生态建设，发展林业经济，继续加快农业产业化步伐。大力推进对俄开放战略升级，积极发展旅游、金融、电业、通信、保险等第三产业。坚持工业立市，多业并举，区域联合，携手发展，以项目建设、招商引资和优化环境为主体措施，推动经济总量快速扩张。全面加强政府自身建设，切实提高政府行政能力，努力开创各项工作新局面。

主要预期目标是：地区生产总值增长12%，财政收入增长10%，全社会固定资产投资增长20%，外贸进出口总额增长20%，利用外资增长20%，城镇居民可支配收入增长8%，农民人均纯收入增长5%，城镇登记失业率控制在4.5%以内，居民消费价格总水平上涨幅度控制在4%以内，社会消费品零售总额增长10%，符合政策生育率控制在94%以上。

政府工作的主要任务是：

(一)以项目建设为载体，加速实施工业立市战略。加快我市的发展，必须坚定不移地实施工业立市的主体战略，必须坚持资源精深加工这个主攻方向，必须突出项目建设这个主要重点。当前，项目建设是经济发展的载体，是经济全局的重中之重，必须强力突破。一是以项目建设为突破口，培育壮大支柱产业。要继续实施振兴老工业基地规划中的“建设两大基地、加快六业发展”的战略。作好煤的文章，加快能源基地建设。要加快鸟山煤矿、新华煤田的建设开发，使鹤矿集团尽快实现年产2000万吨、地方煤矿实现年产500万吨的目标；要用三年时间完成电厂二期60万千瓦工程建设，努力把200万千瓦三期工程列入国家“十一五”计划，争取在“十一五”期间开工建设，实现总装机容量320万千瓦的电力工业规模；完成鹤矿集团10万千瓦矸石热电厂和市政热电联产项目；要加快120 万吨燃料甲醇项目的招商、建设速度，规划建设120万吨焦炭项目，谋划煤层气工业化开采及利用项目，争取短期内在煤化工项目上有大的突破，力争在四煤城抢先建成煤化工基地。作好农牧资源的文章，加快优质食品基地建设。要把农牧资源和食品加工业作为资源型城市转型、发展接续替代产业的战略重点，培育成新的优势支柱产业，争取在三江平原率先建成优质食品工业基地。要加快原料基地建设，建好“第一车间”，保证九三油脂、宝泉岭肉业和全市已有的米业、乳业加工企业达产达效；要整合米业、乳业品牌，形成集团，创建名牌，提高市场竞争力和经济效益；要帮助九三油脂和宝泉岭肉业发展精深加工业，提高技术含量和附加值；要努力扩大奶牛、肉牛养殖规模，引进大型加工企业，把乳业和肉牛加工培育成新的支柱产业；要规划、建设百万立方米规模的林木加工业，尽快实现林木精深加工；要高层次规划、建设石墨加工业，形成主导全国市场的优势产业；要大力发展医药、机械、建材、“两酒”等有资源、有市场、有效益的优势产业。同时要做好180万千瓦的龙江水电站、7亿立方米库容的关门嘴子水库、鹤北至名山铁路、黑龙江大桥、松花江大桥等中远期重大项目的规划、可研工作，争取早立项、早审批、早建设。通过项目建设尽快建成控制资源、占领市场、有效益、有竞争力的优势支柱产业。二是开展项目建设年活动。项目建设决定全市经济发展的全局，今后几年是国家实施振兴东北老工业基地战略的关键时期，也将是我市煤、电、粮、牧、林等主要产业市场较好、效益较好的健康稳定发展期，这是加快项目建设的最佳机遇期。从今年开始，连续三年开展项目建设年活动，就是要在全市提升项目建设的地位，统一对项目建设的思想，明确靠项目出税收、出就业岗位、出经济效益，项目建设决定全市经济全局，决定全市人民的根本利益、共同利益。开展项目建设年活动就是要举全市之力抓项目，锲而不舍、一以贯之地抓项目。要加强领导，集中领导力量抓项目、包项目；要实行项目专项推进机制，做到一个项目一个市级领导、一套班子、一支队伍，一抓到底；要进一步规划调整充实项目，聘请高资质科研机构、专家学者进行项目规划论证和可研工作，进一步优化和充实项目库。力争用三年左右的时间，把我们已经规划的主要项目建成投产一批、开工建设一批、立项待建一批，基本构建成我市的优势支柱产业。三是搞好工业园区建设。遵循经济发展规律，辟建工业园区，集中进行基础设施建设，集中进行管理服务，形成群体效应，降低成本，提高效益。要搞好规划、审批、基础建设和招商引资工作，切实加快园区建设步伐。四是搞好当年项目建设和技改工作。抓好16个重点项目建设，其中鹤电公司二期工程、哈啤(鹤岗)公司10万吨扩产改造等续建项目4项，10万千瓦矸石热电厂、三精千鹤扩产、鸟山煤矿等新开工项目12项，当年计划完成投资7亿元。同时要继续深化企业改革，年内全部完成国有企业的产权制度改革；激活存量，支持华隆玻璃、禾友农药等困难企业调整产品结构，搞活生产经营。2005年，要确保工业增加值增长10%，利税增长20%，地方规模以上企业利税增长30%。

 (二)坚持以农民增收为核心，大力发展农业和农村经济。认真落实中央1号文件精神，全面加强农业和农村各项工作，确保农业增产、农民增收、农村稳定。按照农业工业化、农村城镇化、农村经济产业化的发展方向，加快农业农村经济发展，增加农民收入。要进一步提升农村经济、县域经济在培育支柱产业、支撑全市经济全局、发展接续替代产业和资源型城市转型中的地位和作用。一要提高粮食综合生产能力。优化粮食种植结构，粮食播种面积要达到230万亩，其中水稻面积要恢复到45万亩，粮食总产稳定在4.5亿公斤。推进科技进步，深入实施大豆振兴计划，加快以绥滨县为重点的大豆种植基地建设，专用品种占种植面积的90% 以上。大力推广节本增效、旱作节水等技术，努力降低生产成本，提高农民收益。积极推进标准化建设，全面采用国家标准，制定农业投入品质量标准、农艺技术规程和农产品质量标准，力争在1—2年内使我市农业标准化建设实现重大突破。加强农田水利建设，继续搞好大江大河控制性工程和中小河流治理，重点抓好梧桐河防洪除涝、小鹤立河水库除险加固、松花江绥滨段堤防和元宝山水库建设，搞好黑龙江灌区76万亩水田项目立项准备工作。二要加快发展畜牧业，扩大原料基地规模。要进一步提高畜牧业在农村经济和全市经济中的战略地位，坚持实施“主辅换位”战略，认真落实鼓励畜牧业发展的政策措施，加快畜牧业发展速度，既使农民通过过腹增值增收又为加工企业建好原料车间，促进食品加工业的发展。全市奶牛存栏要达到1.3万头，比上年增长26%；肉牛饲养量9.2万头，增长12%；生猪饲养量56万头，增长33.3%；牧(渔)业增加值达到2.4亿元，增长14.8%，占农业增加值的28%。要从我市农户少、农民人均土地多的市情出发，实施“小区大户”战略，发展专业化、规模化、工厂化养殖基地。引导动员更多的农户从土地中转移出来，从事畜牧业生产；要动员全社会力量参与畜牧养殖，鼓励各类工商企业、合作经济组织和企事业单位兴建一批大规模、高水平、现代化的养殖企业和生产园区，吸引下岗人员、干部职工和社会投资者发展畜牧养殖业。三要进一步发展壮大龙头企业，实现提档升级。要帮助支持九三油脂、宝泉岭肉业等龙头加工企业达产达效、精深加工。要对现有规模比较小、层次比较低的米业、乳业等农畜产品加工企业进行整合升级；要引进建设规模大、竞争力强的名牌加工企业，提升产业化龙头企业水平。四要加强农村社会化服务体系建设。乡镇机构改革后，要进一步调整转换职能，强化对农民和农村经济的服务，为农民和企业提供信息、技术、政策等各项服务，促进农村经济发展，搞好县乡财源建设。同时要大力发展农村中介组织，用市场经济的办法解决农村经济发展中的问题。今年要实现农业增加值8.64亿元，比上年增长8%。

(三)深化经营城市改革，进一步加快城市发展。要运用市场经济的理念和手段，深化经营城市改革，拓宽城市建设的投融资渠道，进一步提高城市建设和管理水平，增强和完善城市功能，促进城市经济的快速发展。一是搞好沉陷区治理二期工程和城市新区建设，扩大住宅建设规模。今年要启动沉陷区治理二期住宅建设工程，开工建设60万平方米，其它住宅建设要实现开竣工50万平方米。要考虑我市城市大拆迁、大建设已连续八年，城市楼、平房比例发生很大变化和拆迁中遇到诸多突出矛盾的实际，结合沉陷区二期治理的需要，按照控制拆迁规模、降低拆迁成本、减小拆迁损失和拆迁难度、照顾平房区困难户利益，特别是保持楼房、平房合理比例、逐步扩大建成区规模的原则，按照城市总体规划，辟建城市新区，加快城市发展。要切实解决城市建设中拆迁难的问题，进一步调整拆迁政策，保护被拆迁户的合法权益，保证依法拆迁、城市建设的顺利进行。二是加快基础设施建设。新、改、扩建5条城市主次干道。继续推进“五化”建设和“十小工程”，加快城市排水、防洪工程建设。抓好绥嘉公路绥滨至名山段、哈肇公路鹤岗至名山段复线、鹤北支线等工程建设；完成通乡、通村公路建设任务，力争突破300公里。争取建设货运枢纽工程。三是进一步提高城市管理水平。建立完善城市管理的长效机制，加大综合执法力度，突出抓好综合治理。按照建设生态园林城市的要求，搞好城市净化、绿化。推进城市管理体制、机制创新，着力降低城市管理成本，提高管理效益。四是积极探索经营城市的新方式、新办法。大力推行经营性用地招标、拍卖、挂牌出让制度，开放土地二级市场，全面实行土地有偿使用，推动土地按市场机制流转和升值；加强土地储备，推行土地收购储备制度，实施集约供地。积极推进市政公用设施建设、经营、管理的市场化运作，通过拍卖所有权、经营权等筹集城市建设和管理资金。

(四)加快推进南联北开，实现对俄经贸合作战略升级。充分发挥区位优势，以实现对俄经贸合作战略升级为重点，推进对外开放，进一步开发利用国内外两种资源、两个市场。以对俄合作为重点，加速实施南联北开战略，提高整体经济外向水平，确保口岸过货量达到20万吨，过境人数达到2万人次，分别增长17.6%、5.3%。一是进一步发挥驻俄商务代表处作用，搞好对俄合作的协调联系和指导服务。配齐配强驻俄商务代表处工作力量，密切与俄方的沟通协调，确保双方联系渠道畅通；积极为国内企业提供信息和项目，加强对我方赴俄企业的指导服务，扩展合作发展空间；筹划组建驻俄华人商会，维护企业合法权益。二是组织市内企业加大对俄方市场的开发力度。要继续坚持以开发俄方土地、森林、矿产资源为重点，帮助市属企业和鹤矿集团、宝局、鹤北局等大企业扩大在俄方的投资、开发规模。要充分利用我市同俄方互补性强的优势，引导组织我市现有建筑、商贸、油米加工企业开发俄方市场，积极拓展对俄资源和市场开发的领域和深度，切实提高效益。三是南联国内发达地区有实力企业，北开俄方内陆市场。对俄经贸合作必须突破我市企业规模小、实力弱，俄犹太州人口少、市场小的局限，在南联国内发达地区大企业和通过俄犹太州向其内陆地区拓展上有大的突破。充分发挥我市与俄方关系友好的桥梁纽带作用，引进南方有实力企业，到俄方从事经贸、生产加工、资源开发等产业，把对俄经贸合作在领域、层次和水平上向纵深推进。四是加强对俄合作项目的申报立项和向上争取。今年国家和省都把对俄经贸合作做为投资支持的重点，我们要抢抓机遇，积极向上争取，在口岸建设、进出口加工基地建设、对俄资源开发重大项目以及建设互市贸易区、界江大桥、名山铁路等基础设施项目上获得更多的支持，有更大的进展。

(五)大力发展非公有制经济，增强整体经济实力。我市原国有工商企业改制后成为民营企业，非公有制经济已经成为我市地方经济的主体，必须坚定不移地促其加快发展、做强做大。今年要实现非公有制经济增加值25.3亿元，增长16%，实现税收1.44亿元，增长13.4%。一是引导非公有制企业机制创新，建立现代企业制度。我市非公有制企业多数规模小、层次低，发展到一定阶段后，管理方式和企业素质不适应更快发展和激烈的市场竞争需要，要通过学习培训、典型示范等手段，引导非公有制企业完善法人治理结构，采取先进的管理方式，重视重用人才，逐步建立现代企业制度。二是引导支持非公有制企业拓展经营领域，做大做强骨干企业。我市民营企业多集中在煤炭生产、房地产开发、餐饮服务、油米加工四大领域，多数企业和行业市场空间小，要引导和支持民营企业优化经营结构和投资结构，靠紧我市经济发展战略，向能源食品加工领域、畜牧养殖、城市基础设施、社会公益事业、各类加工业、高科技领域和开发俄罗斯资源、市场转移，拓展生存发展的新空间。要引导支持企业在市内企业之间、市外知名大集团大企业之间挂靠联合，提高素质，做强做大。在全面促进民营企业加快发展的同时，今年要重点抓好万禾元油脂、华盛木业、人和粮业、泰丰米业、峻丰洗煤厂、鲲鹏焦化6个项目的建设和达产达效。三是进一步加强对非公有制经济的服务、扶持。切实在市场准入、信贷、税费、投融资等方面创造平等竞争的条件。加强产业引导，强化项目、技术、信息、融资等社会化服务。

 (六)以旅游业和物流业为重点，加快发展现代服务业。一是大力发展旅游业。要抢抓国内旅游市场旺盛、我省旅游业发展加快的机遇，依托我市旅游资源，融入旅游市场的分工合作和竞争，加快我市旅游业的发展。要坚持森林、界江、冰雪、境外资源的综合开发，突出黑龙江三峡界江游的特色，叫响“不到黑龙江不算到龙江，要到黑龙江最近最美是鹤岗”的品牌，逐步形成“南海北江”特色游。继续推进旅游重点项目建设，建设太平沟景区的道路、服务设施等基础设施。筹建龙江三峡旅游车船公司，开通名山——嘉荫河口的旅游航线，形成鹤岗——龙江三峡——嘉荫——伊春的旅游大环线，打造高等级的黄金旅游线路。二是加快发展现代物流业。要适应对俄开放升级、旅游业发展和我市经济加快发展的需要，加快发展现代物流等第三产业，切实为经济发展提供优质服务，又使第三产业成为市域经济重要支柱。

(七)硬化工作措施，实现招商引资新突破。一是要提升招商引资的地位，壮大工作力量，实行全民招商。招商引资是加快经济发展的主体措施，是我市的出路和希望所在。要把招商引资做为全社会、全民的共同责任，形成全社会、全民招商的合力。市级领导要分工负责重点项目招商；市直各单位和全体干部职工要全部参与招商引资，实行目标责任管理；实行强力激励机制，重奖招商引资有功人员，动员全民招商。市政府设立专项奖励基金，用于兑现招商引资奖励政策。二是要突出招商引资重点。在全面推进招商引资工作的同时，重点突出在市场上的项目招商和对上对外的引资，从今年起市政府建立项目招商和对上对外引资两项重点工作专项考核奖励办法，激励招商引资做实事、求实效。要把招商引资主攻方向集中到我市规划的主导产业主要项目的同类产业、同类企业聚集区、国内外大企业集团、国内外经济发达地区和各地各级各类经济开发区，力求走捷径、见成效。三是要创新招商方式，硬化政策措施。要总结近年来招商引资工作经验，在坚持有效办法的同时创新方式和政策措施。要进一步坚持完善招商引资优势共享政策，形成全区域招商合力；要进一步完善和兑现招商引资激励奖励政策，调动全民全社会招商积极性，形成全民招商、全社会招商的态势；要实行专项定向招商工作机制，对重点项目组织专门领导、专门班子、专门队伍、专业力量、一招到底的专业项目招商组；组织优秀干部到重点经济开发区、省招商局挂职定向招商；要更多地参与国家和省组织的各种招商活动，力求有大成效。

(八)强化政府协调服务职能，推进区域经济联合协作。板块经济是我市基本市情，鹤矿集团、宝局、鹤电公司、鹤北局都是我市区域内经济实力强、发展速度快、潜力活力大、国家支持多的经济主体，激发各经济单元的发展活力是市域经济发展的工作重点。要发挥市县政府的职能功能优势，加大对这些经济单元的协调服务力度，整合资源和力量、形成经济社会发展合力，在加快发展中实现共荣共赢。一是要在发展战略上形成合力。“两大基地、六大产业”要成为域内共同的战略。二是要在项目开发上形成合力。围绕域内规划的项目，共同在市场招商，共同向上争取支持。三是要在利用开发资源上形成合力。坚持不求所有、但求所在的原则，共同开发利用资源，实现政府增税、企业增利、职工群众增就业、兴旺一方事业的共同利益。

(九)做好财税金融工作，支持经济社会快速发展。一是要加大争取上级支持的力度，加快财源建设。要充分利用国家振兴东北等老工业基地战略和省实施市县财源建设战略的机遇，争取更多的政策、项目、资金支持，促进市县(区)财源建设，不断增加各级财力。二是要加大税收征管力度，确保财政税收计划完成。要坚持依法治税、公开办税和财政改革方向，加大税收征管力度，强化重点税源和零散税源的征管，努力做到应收尽收，确保财政税收计划的圆满完成。三是要增收节支，确保预算平衡。要坚持过紧日子的思想和勤俭节约的方针，严格控制财政支出，强化措施，堵塞漏洞，确保工资发放和重点支出，确保财政收支平衡。四是要支持金融机构健康运行。支持金融机构优化信贷结构、降低不良资产比例、防范金融风险，更好地为地方经济建设和发展服务，努力解决好民营企业、中小企业融资难的问题。要深化农村信用社改革，在全面完成增资扩股任务的基础上，理顺管理体制，转换经营机制。

(十)大力实施科教兴市、人才强市战略，强力推进科技进步。科技是经济的先导，教育是基础，人才是发展的支撑，今年要着重抓好人才的培养、吸引和使用三个环节，提高科技研发能力，提高科技普及率，进一步提高科技、人才对经济发展的贡献率。一是要实行特殊优秀拔尖人才的优惠政策。对于优秀高级工程技术人员、管理人员、医护人员、教师等拔尖人才，在工资、福利、待遇、录用(聘用)管理上要给予特殊优惠，营造引进、留住和使用人才的优良环境。要建立优秀拔尖人才奖励机制，建立优秀拔尖人才奖励基金，提高对人才的待遇和奖励水平。二是要加强本土人才培养。多渠道、多途径培养适应产业调整和发展需要的适用型人才，加大对经济发展急需的专门人才的培养力度。三是要促进科技成果转化。加强与大专院校、科研院所的对接，积极引进专家学者的智力成果，在重点行业、企业、产品上加大高新技术成果的引进和应用，更好地为企业提供科技信息服务。要加快高新技术产业和民营科技企业发展，提升产业层次和水平。四是要抓好教育事业。按时完成教育人事制度改革，改革学校内部管理体制和分配机制。巩固基础教育成果，进一步加大教育投入，改善办学条件，加大危房改造力度，继续实施学校网点布局调整三年规划。大力发展职业教育，为我市经济社会发展培养实用型人才。办好鹤岗师范专科教育，提高自我发展能力。加快发展农村教育和民办教育，促进全市教育的均衡和全面发展。

 (十一)坚持以人为本，切实解决好人民群众关心的热点难点问题。要坚持以人为本的科学发展观和正确的政绩观，在加快发展的同时，把提高人民生活水平放在各项工作的首位，努力为人民排忧解难、办好事、办实事。一是要继续做好社保试点工作。严格贯彻执行相关政策，积极筹措匹配资金，确保完成并轨任务；要调整和完善基本养老保险制度，把做实个人账户规模由5%提高到6%；要抓好社会保险扩面工作，把重点放在灵活就业人员上；要加强城镇居民低保动态管理，努力实现应保尽保。切实抓好农村低保试点和医疗救助工作，逐步完善农村社会保障制度。二是要努力扩大就业和再就业。要加快项目建设，增加就业；要引导下岗失业人员更新就业观念，在就业岗位多的服务业、建筑业、煤炭生产、农业、畜牧业等领域创业就业。要巩固劳务输出品牌，继续向经济发达城市输出合格劳动力，减轻就业压力。抓好公益性就业岗位开发管理，帮助就业困难群体实现再就业。要加强技术培训工作，提高就业能力。全年要完成就业再就业2.1万人。三是要加大依法维权力度。努力营造依法用工、依法管理、依法维护劳动者合法权益的氛围，实行部门联动，切实解决好拖欠职工尤其是农民工工资问题。四是要强化便民举措。全面提高市民医、食、住、行等公益事业的服务水平。整合资源，实行“119、120、110”三网合一，为群众提供便捷服务。要进一步搞好街道、社区建设，提高为民服务水平，打牢社会安定稳定基础。要畅通信访渠道，倾听群众呼声，认真解决群众来信来访提出的问题。四是要合力做好扶贫解困工作。坚持雪中送炭，更多地关心社会贫困和弱势群体的生产生活，切实做好扶贫解困和救助工作，维护好人民群众的根本利益。

(十二)大力发展各项社会事业，努力构建和谐社会。要以建设“文明鹤岗”为目标，全面推进各项社会事业的发展。加强公共卫生体系建设和医疗基础设施建设，健全医疗救治体系，加快社区卫生服务示范区建设，全面建立社区卫生服务网络；搞好绥滨农村合作医疗试点工作；强化农村卫生工作，改善基础设施，完善服务功能，提高医疗水平。巩固人口与计划生育工作成果，继续保持全省领先水平。加快文化体制改革步伐，适时启动文艺团体改革；繁荣文化产业和文化事业，努力打造艺术精品，丰富群众文化生活。要积极吸引社会资金参与广电事业发展，推进数字化进程。深入开展全民健身运动，抓好竞技体育发展，继续保持在全省先进行列。加强环境保护工作，进一步搞好大气污染治理、水源保护和退耕还林、还草、还湿工作。深入实施“平安工程”，整合警力资源，建立新型动态警务模式；健全社会治安防控体系，形成高密度、网络化的防控格局；严厉打击各种犯罪，大力整治突出问题，完善严打长效机制，提高治安防控能力，创造“平安鹤岗”。认真落实安全生产责任制，采取有力措施，实现矿井投资人与法人代表的统一，让投资人真正负起安全生产责任；以“一通三防”为重点，抓好煤矿安全专项整治，增加安全设施投入，加强安全监管，落实领导责任，杜绝3人以上重特大事故的发生，地方煤矿百万吨死亡率控制在2.4以内，消防、交通、危险化学品等各项安全要强化措施，抓好落实，确保不发生重大事故。大力整顿和规范市场经济秩序，重点抓好食品、药品的市场监督，严厉打击制造销售假冒伪劣食品、药品行为；严肃查处药品购销中的不正之风，大力推行公共采购，从源头上保证采购药品的质量。要进一步做好民族、宗教、外事、侨务、国防、人防、民政、双拥、人事、档案、老龄等各项工作。

三、加强政府自身建设

我市加快发展的新机遇、新形势、新任务，向政府工作提出了全新的要求，政府必须全面履行经济调节、市场监管、社会管理和公共服务职能，进一步加强自身建设。

（一）要深度解放思想，锐意开拓进取。要在加快发展上进一步解放思想。政府全体工作人员都要牢固树立竞争图强、加快发展的理念，要充分认识到加快发展是解决人民群众各种困难和问题的根本出路，是全市共同的大局，是硬道理，是第一要务；要实现加快发展，我们确有困难，但也有机遇和条件。要在欠发达地区定位上进一步解放思想。我们要敢于面对现实，清醒准确认识我们在国内、省内处于欠发达地区的市情，在全国、全省各地竞相发展的激烈竞争和发达地区经济科技实力强大压力下，要实现快发展，就要坚持实行比别人更开放、更优惠的政策，更过硬、更有效的措施，更宽松、更优良的环境，更加艰苦、更多付出的努力，因而要坚持与时俱进、大胆改革创新。

（二）要加强学习和实践，提高驾驭市场经济和解决实际问题的能力。政府全体工作人员都要把提高驾驭市场经济和解决实际问题的能力，作为自身学习、提高的重点，切实加强对政治理论、政策法规、科学知识、经济规律、先进经验、群众智慧的学习，加强对市情、对本职工作、对阻碍发展进步的实际问题和实践中层出不穷的新情况新问题新矛盾的研究，力求在理论和实践的结合上，在解决实际问题的能力上，在推进经济快速发展上有大的提高、大的作为。

（三）要建设求真务实、有效落实的过硬作风。要实现加快发展的宏伟目标，任务艰巨，工作量大，要求我们必须有真抓实干、有效落实的过硬作风。要切实树立和坚持科学发展观和正确政绩观。一切为了发展，一切为了人民，一切为了工作实效，不搞短期行为，不搞政绩工程、形象工程，不搞虚假浮夸，不搞形式主义，强化“讲真话、办实事、求实效”的作风，在全力推进经济社会快速发展的同时，肯下硬功夫，打基础，攒后劲。要敢于碰硬，勇于克服困难，解决问题。面对经济社会发展和人民群众生产生活中的大量困难、矛盾和问题，我们必须迎着困难上，不回避矛盾和问题，遇到什么困难就致力于克服什么困难，出现什么问题就努力解决什么问题，真正做到负责高效。要深入实际，狠抓落实。要深入基层，深入群众，深入实际，狠抓工作落实，一定要树立求真务实、狠抓落实、一抓到底、讲求实效的工作作风。

(四)要坚持清正廉洁，建设廉洁政府。政府及其工作人员必须以廉洁政府和人民公仆作为自身的工作目标和行为准则，要认真落实党风廉政建设的各项要求，切实搞好政府自身廉政建设。要从严治政。要坚持从严治政的方针，强化政府部门的队伍建设；要继续加强行风评议，以经济管理部门、行政执法部门、公用事业单位和人财物管理人员为重点，抓住群众反映强烈、影响改革发展的突出问题进行专项治理，提高政风行风水平。要廉洁从政。政府全体工作人员要严格按照廉洁自律的要求规范言行，带头遵纪守法。各级领导干部都要严格遵守《廉政准则》，严格要求家属、子女和身边工作人员，做廉洁自律的表率，用清正廉洁的良好形象取信于民。要深入开展警示教育和保持共产党员先进性教育，严厉查处腐败案件，建立健全长效机制。

(五)要坚持改革创新，促进政府职能转变。要围绕建设法制政府、廉洁政府、阳光政府、高效政府、务实政府、有为政府、为民政府、服务政府、诚信政府的目标，深入探索和创新有治本作用、长效作用的机制制度，促进政府职能转变，提高政府工作水平。一要进一步改革行政审批制度，坚持依法行政。要进一步减少行政审批事项，向企业、市场和中介组织放权；要认真实施《行政许可法》和《全面推进依法行政实施纲要》，进一步开展学法活动，不断增强政府公务人员的法制观念，严格按法定权限和程序行使权力、履行职责，自觉用法律手段进行经济社会管理。二要实行公开办事和公示制度，推进阳光政务。各级政府要把政务公开作为基本施政制度，除特殊机密性事务外，能公开的一律向社会、向群众公开、公示，自觉接受人民群众和全社会的监督。三是重大决策实行听证论证制度，实行科学民主决策。对经济社会发展的重大事项、重要决策，对涉及人民群众切身利益和社会公共利益重点难点问题的处理，都要组织有关专家、各界人士、群众代表和相关人员参加的论证听论，广泛听取专家学者的意见和人民群众的意见、建议，进一步扩大人民群众对政府事务的参与程度，集思广益，民主科学决策。四是实行部门述职评议制度，强化社会监督。今年要有一些部门接受人大的述职评议，自觉接受人大和政协的法律监督、民主监督。不定期推出一些人民群众关注的部门进行社会民主评议，依靠群众和社会的力量治理痼疾顽症。要进一步搞好行风评议工作，提高全社会监督的效果和质量。五是实行集中办事、统一开放管理制度，切实方便群众，管好队伍。在过去一站式服务机制的基础上，今年建设启动“人民办事中心”，凡涉及人民群众到政府办事的都纳入中心，集中办事，一站式服务。进入办事中心的政府部门对人民群众的办事要求实行中心内受理，中心内办结，中心对进驻人员实行统一管理和吸纳各界代表参加监督的开放式管理，严明纪律，提高效率。六是实行政府新闻发言人制度，扩大与人民群众的沟通联系。对政府重大决定事项、经济社会发展的主要情况和人民群众关注的重点难点问题，不定期进行新闻发布，及时通报情况，提高政府工作的透明度，扩大人民群众的知情权，畅通政府与人民群众的联系渠道，增进了解，加深理解，形成合力。七是实行政府分级协调服务制度，切实提高服务效率和水平。对企业和人民群众生产生活中存在的问题和要求，每季度要由政府有关部门搜集整理一次，政府办汇总后向政府职能部门、主管副市长和市长三个层面通报汇报，自下而上分阶段限期研究解决问题。八是对政府工作人员实行全员目标责任管理制度，完善工作机制。政府各部门对全年的任务目标和工作职责要细化量化到单位、到岗位、到人头，做到人人有事干、事事有人管、横向到边、纵向到底。同时要建立明确可行的奖罚措施，及时考核兑现，解决机关工作实际中存在的大锅饭问题，确保政府各项工作和任务落到实处。九是对执法执罚人员实行专项纪律管理制度，创建优良发展环境。对经济环境有重大影响的执法执罚人员实行专项纪律管理，对违法违纪行为制定明确严格的处罚办法，严格执行。十是建立督办督查和责任追究制度，强化工作落实。对政府决策和部署的各项工作，要建立严格的督办督查制度，及时通报。对出现问题、造成后果的，要依法依纪严肃追究。以上十项制度政府将制定专项办法具体落实，同时要进一步坚持和完善工程招投标、土地挂牌出让、财政收支两条线、政府统一采购、国库统一支付等各项改革措施。

各位代表，今年是我市抢抓机遇、加快发展至关重要的一年，任务艰巨，责任重大，让我们高举邓小平理论和“三个代表”重要思想伟大旗帜，在以胡锦涛为总书记的党中央和省委、省政府、市委的正确领导下，奋力工作，合力攻坚，全力推进经济社会的快速健康发展，全面完成市委工作会议和这次人代会确定的各项任务，为建设边疆名城、提高人民生活水平做出新的贡献!
